# Etiologies and Management of Aseptic Meningitis in Patients Admitted to an Internal Medicine Department

**DOI:** 10.1097/MD.0000000000002372

**Published:** 2016-01-15

**Authors:** Irène Jarrin, Pierre Sellier, Amanda Lopes, Marjolaine Morgand, Tamara Makovec, Veronique Delcey, Karine Champion, Guy Simoneau, Andrew Green, Stéphane Mouly, Jean-François Bergmann, Célia Lloret-Linares

**Affiliations:** From the Assistance Publique Hôpitaux de Paris, Unit of Therapeutic Research, Department of Internal Medicine, Hôpital Lariboisière, Paris, France (IJ, PS, AL, MM, TM, VD, KC, GS, SM, J-FB, CLL); and Yorkleigh Surgery, Cheltenham, Gloucestershire, UK (AG).

## Abstract

Several studies have focused on the clinical and biological characteristics of meningitis in order to distinguish between bacterial and viral meningitis in the emergency setting. However, little is known about the etiologies and outcomes of aseptic meningitis in patients admitted to Internal Medicine.

The aim of the study is to describe the etiologies, characteristics, and outcomes of aseptic meningitis with or without encephalitis in adults admitted to an Internal Medicine Department.

A retrospective cohort study was conducted in the Internal Medicine Department of the Lariboisière Hospital in Paris, France, from January 2009 to December 2011. Clinical and biological characteristics of aseptic meningitis were recorded. These included cerebrospinal fluid analysis, results of polymerase chain reaction testing, final diagnoses, and therapeutic management.

The cohort included 180 patients fulfilling the criteria for aseptic meningitis with (n = 56) or without (n = 124) encephalitis. A definitive etiological diagnosis was established in 83 of the 180 cases. Of the cases with a definitive diagnosis, 73 were due to infectious agents, mainly enteroviruses, Herpes Simplex Virus 2, and Varicella Zoster Virus (43.4%, 16.8%, and 14.5% respectively). Inflammatory diseases were diagnosed in 7 cases. Among the 97 cases without definitive diagnoses, 26 (26.8%) remained free of treatment throughout their management whereas antiviral or antibiotic therapy was initiated in the emergency department for the remaining 71 patients. The treatment was discontinued in only 10 patients deemed to have viral meningitis upon admission to Internal Medicine.

The prevalence of inflammatory diseases among patients admitted to internal medicine for aseptic meningitis is not rare (4% of overall aseptic meningitis). The PCR upon admission to the emergency department is obviously of major importance for the prompt optimization of therapy and management. However, meningitis due to viral agents or inflammatory diseases could also be distinguished according to several clinical and biological characteristics highlighted in this retrospective study. As recommendations are now available concerning the prescriptions of antiviral agents in viral meningitis, better therapeutic management is expected in the future.

## INTRODUCTION

Several studies have focused on the clinical and biological characteristics of meningitis, in order to distinguish bacterial from viral meningitis and to provide the suitable treatment.^1–3^ Bacterial meningitis is life threatening and requires prompt administration of antibiotics whereas viral meningitis often improves without curative treatment. Antiviral therapy has changed the prognosis of herpes simplex virus (HSV) encephalitis, but excessive prescriptions can lead to increased toxicity and avoidable medical expenses.^4,5^ As the clinical picture of bacterial and viral meningitis are similar, the analysis of cerebrospinal fluid (CSF) is essential in order to distinguish them, identify the virus, and decide whether or not to initiate antiviral therapy.^5^ Polymerase chain reaction (PCR) identifies the most commonly involved viruses: Enterovirus, HSV, and Varicella Zoster Virus (VZV).^1–3,6^ However, despite the progress in diagnostic methods, the cause is identified with certainty in only 30% to 65% of aseptic meningitis.^1,2^

The prevalence, clinical, and biological pictures of causes that are more rare than viral pathogens have rarely been reported. Previous studies focused either on the clinical pictures of a particular type of bacterial or viral meningitis, or on a specific population, usually in a pediatric or emergency setting.^7–11^

We therefore decided to conduct a retrospective study to describe the etiologies, characteristics, and outcome of aseptic meningitis with or without encephalitis in adults admitted to an Internal Medicine Department.

## METHODS

### Patients

We conducted a monocentre study at the Internal Medicine Department of the Lariboisière Hospital in Paris, France. Records of patients admitted for meningitis in our Department were retrospectively extracted from the Programme de Médicalisation des Systèmes d’Information (PMSI) data processing centre. This database is a national register of all discharges from all short-stay/acute-care hospitals. It collects data described by the physicians who took care of the patients during their hospitalization, using the International Classification of Diseases, 10th revision (ICD-10). We included all adult patients (aged 16 years or older) admitted for meningitis (with white blood cells [WBC] >5/mm^3^ in CSF) between January 2009 and December 2011. Patients with purulent or positive direct stain meningitis were excluded from the study.

The same investigator reviewed the medical records of all screened patients. The following clinical and biological data were recorded: demographic characteristics (gender, age), headache, fever > 100.4 °F (38°C), neurological examination, CSF analysis, and liver functions (aspartate aminotransferase, AST; or alanine aminotransferase, ALT). Therapeutic data were also recorded: prescription of antiviral drugs or antibiotics, length of prescription, and the type of antibiotics.

This study followed the Declaration of Helsinki on medical protocol and ethics and the regional Ethical Review Board of the Northern University Hospital Group of Paris approved the study.

### Diagnosis

A lumbar puncture was performed in all patients. They were suspected of having encephalitis, associated with acute meningitis (AM), if they displayed signs or symptoms suggestive of cerebral involvement: seizures, reduced consciousness, confusion, or focal neurologic signs. According to clinical guidelines, these patients underwent computed tomography and/or magnetic resonance imaging of the brain was performed before lumbar puncture in patients with suspected encephalitis or intracranial abnormality, such as history of central nervous diseases.^12,13^

### Etiological Diagnosis of Aseptic Meningitis

A polymerase chain reaction testing was performed from CSF samples for the identification of HSV-1 and -2 or VZV [RealStar alpha Herpesvirus PCR Kit1.0 (ALTONA)] and Enterovirus [geneXpert Enterovirus Xpert (Cepheid)], irrespective of clinical presentation or WBC count in the CSF. No exploration other than PCR to look for enterovirus was performed during the period study. A second lumbar puncture was discussed with the patient if the investigations at admission were incomplete. Additional investigations were performed in immune-compromised patients, or based on the clinical examination: PCR for cytomegalovirus (CMV) [Abbott RealTime CMV], John Cunningham Virus (JCV) [RealStar JCV PCR kit 1.0 (ALTONA)], Epstein-Barr virus (EBV) [EBV Qiagen M2000sp-M2000rt V4 (Abbott)], Human Herpes Virus 6 (HHV-6) [RealStar HHV-6 PCR Kit (ALTONA)], Toxoplasma gondii [RealTime, Applied Biosystems 7500] and Mycobacterium tuberculosis [kit MTB/RIF (Cepheid)], Indian ink test for Cryptococcus. No additional exploration was prescribed in the case of fast improvement.

The circumstances that lead to additional exams were abnormalities in clinical or biological assessment (sign of encephalitis, weight loss, alteration in general state, abnormalities in the clinical exam, persistence of abnormalities in further CSF analysis) or an abnormal short or long-term evolution. The management of the encephalitis was guided by French guidelines.^14^ According to the clinical situation, additional explorations were performed in blood (Lyme borreliosis, syphilis, rubella, measles virus serology) and in CSF (PCR for Detection and Characterization of HHV-8, mycobacterium tuberculosis, BK virus, parvovirus B19, *Neisseria meningitidis*, *Haemophilus influenzae*, and *Streptococcus pneumoniae*, Lyme borreliosis or Syphilis serology, HIV viral load, CSF opening pressure, Cryptococcus Antigen, CSF angiotensin-converting enzyme). When an autoimmune disease was suspected, the autoimmune biology included the research of 1 or several antibodies: antineutrophil cytoplasmic, antinuclear, antidouble-stranded DNA, antiextractable nuclear antigen, antithyroid, anti-Hu, anti-Yo, anti-Ma, anti-Tr, anti-N-methyl D-aspartate receptor.

### Statistics

Statistical analysis was performed using Statview v 4.0 (SAS Institute, Cary, NC). Quantitative data were presented as median and minimum–maximum values and were compared using nonparametric Mann–Whitney tests. Fisher's exact tests were performed to compare categorical values. A *P* value of 0.05 or less was considered significant.

## RESULTS

**FIGURE 1:**
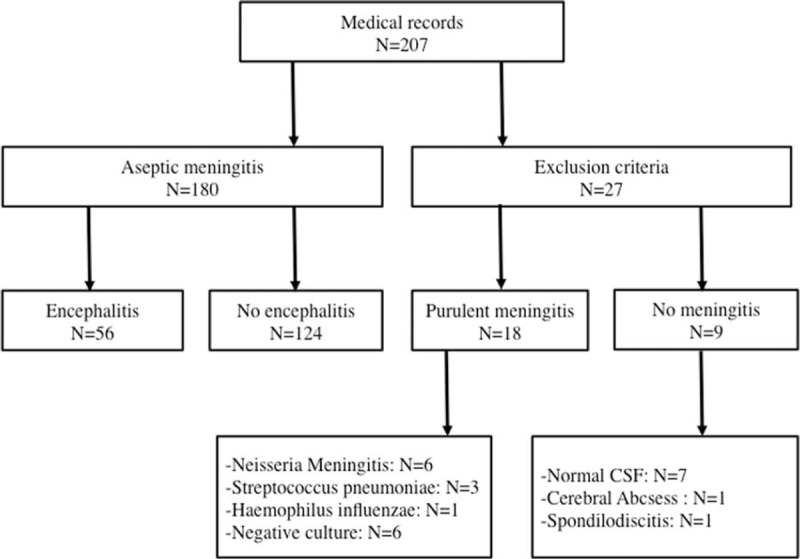
Study flowchart.

Figure 1 shows the flowchart of the study. Of the 207 medical records of meningitis extracted from the hospital database, 180 patients fulfilled the criteria for AM with (n = 56) or without (n = 124) encephalitis. No patients had >1 episode of AM during the retrospective study period.

### Clinical and Biological Characteristics of the Population

The population's clinical and biological characteristics are presented in Table 1. All clinical symptoms were less frequent in patients with encephalitis compared to patients without encephalitis (headache: n = 44[78.6%] vs n = 124[100%]; fever: n = 19[33.9%] vs n = 65[52.4%]; neck stiffness: n = 20 [35.7%] vs n = 66[53.2%]; nausea/vomiting n = 13[23.2%] vs n = 62[50.1%], *P* < 0.005). Skin and mucosal lesions were observed in 33 cases (18.3%) and the frequency did not differ significantly between patients with or without encephalitis. Encephalitis symptoms were cranial nerve palsy in 18 cases (32.1%), other focal neurologic sign (paresthesia, dysesthesia, and hemiparesis) in 9 cases (16.1%), confusion in 8 cases (14.3%), spatial and temporal disorientation in 6 cases (10.7%), seizures in 5 cases (8.9%), and other symptoms (cognitive impairment, motor apraxia, and cerebellar syndrome) in 11 cases (19.6%).

**TABLE 1:**
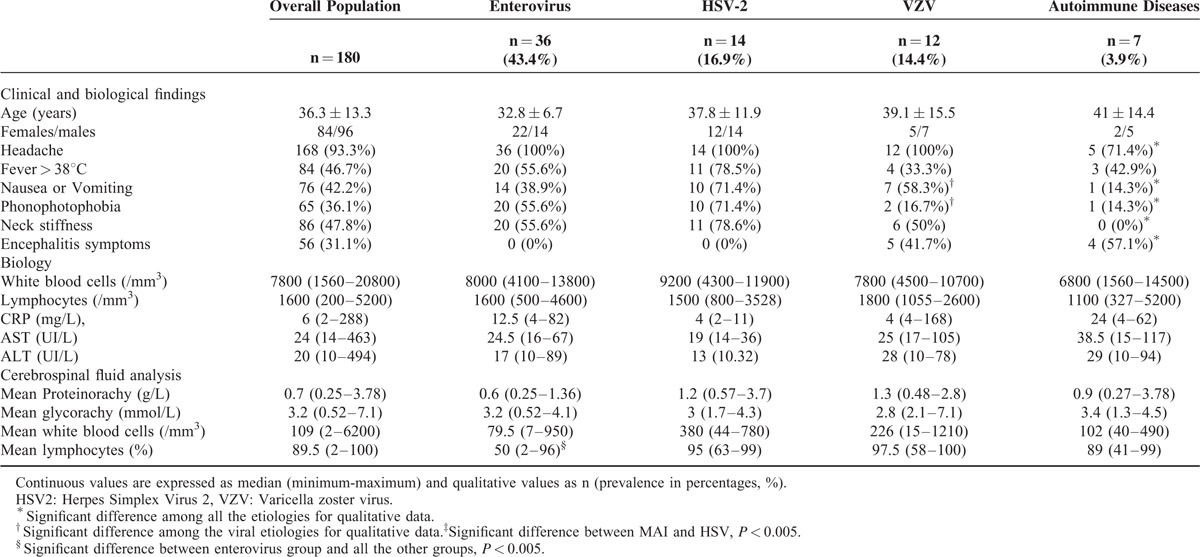
Characteristics of Aseptic Meningitis and of Their Main Etiologies

CSF protein was higher in patients presenting with encephalitis (median: 0.78, range: 0.27–3.8 vs median: 0.7, range: 0.25–3.7, *P* < 0.005).

### Meningitis/Encephalitis With Certain Diagnosis

Among the 180 cases of AM, a definitive etiological diagnosis was found in 83 cases (46.1%) and 73 cases were due to the infectious agent.

#### Characteristics of Infectious Aseptic Meningitis

The most common microorganisms were enteroviruses (43.4% of AM with a definitive diagnosis), HSV-2 (16.8% of AM with a definitive diagnosis), and VZV (14.5% of AM with a definitive diagnosis). The clinical and biological findings of each virus are presented in Table 1. We compared clinical symptoms, biology, and CSF analysis among the main infectious causes of AM. Among the clinical findings, only nausea/vomiting and phono-photophobia were found significantly more often in patients with AM due to HSV-2 than in patients with AM due to enteroviruses or VZV.

**FIGURE 2:**
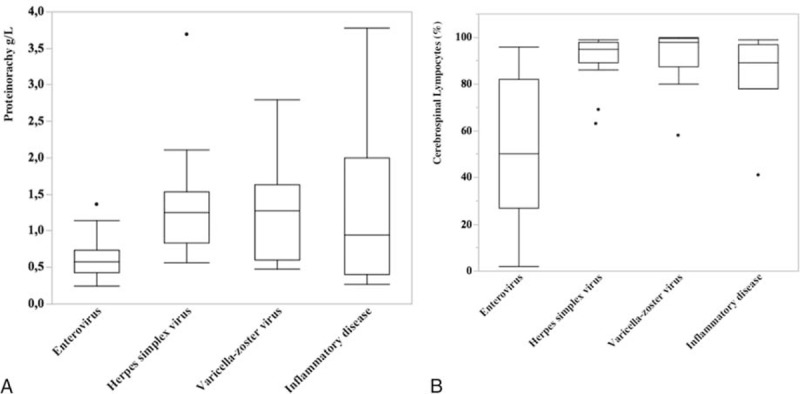
Comparison of cerebrospinal fluid analysis between the main etiologies of meningitis: lymphocytes in percentage (A) and proteinorachy in g/L (B).

Patients with AM due to HSV-2 were mainly women (86%). Five of the 14 cases (36%) had clinical genital herpes at admission, and 1 had oral herpes. The meningitis was a (fourth) recurrence in 1 case. Of the 12 patients with AM due to VZV, 4 had simultaneous herpes zosters (1 of them, who was immune compromised, showed a negative PCR). Among the 4 cases with encephalitis, 2 had simultaneous zosters. Physical signs of infection occurred before AM in 15/36 cases (41.7%) of meningitis due to enteroviruses, either in the patient, or in the people who were in contact with the patient. Signs of upper respiratory tract infection were the most common. The CSF protein level of patients with AM due to enterovirus was significantly lower than in patients with AM due to HSV-2 or VZV (Table 1, Figure 2A). The lymphocyte percentage in the CSF of patients with AM due to enterovirus was significantly lower than in patients with AM due to HSV-2 or VZV (Figure 2B).

The other diagnoses of infectious meningitis were: tuberculosis (n = 4), leptospirosis (n = 1), toxocariasis (n = 1), cysticercosis (n = 1), cryptococcosis (n = 1), lyme disease (n = 1), acute HIV infection (n = 1), and listeria monocytogenes (n = 1).

#### Characteristics of Noninfectious Aseptic Meningitis

Inflammatory diseases were diagnosed in 7 cases (4% of AM, 8.4% of AM with a definitive diagnosis): 2 men, 28 and 59 years old, with Behcet's disease; a man aged 46 and a woman, aged 59 with sarcoidosis involving the CNS, a 29-year-old woman with acute systemic lupus erythematosus (SLE) with psychiatric manifestations; a 41-year-old man had Kikuchi disease, and a 25-year-old man had Hashimoto encephalopathy. In each case, the diagnosis was unknown before the investigations in internal medicine. A comparison of the characteristics of the 3 main infectious etiologies with those of inflammatory diseases is presented in Table 1. The respective clinical and biological features of each case are presented in Table 2. In comparison with viral meningitis, the clinical picture of meningitis due to inflammatory diseases was frequently incomplete: none of the patients had neck stiffness, whereas headache, nausea or vomiting, and phono-photophobia were significantly less frequent. Encephalitis symptoms were the main clinical symptoms at admission, found in 4 of the 7 patients. The variability in the protein level (median: 0.9 g/L, range: 0.27–3.78 ) was considerable and higher than in enterovirus meningitis (Figure 2A). The lymphocyte percentage in CSF was high and similar in range to those with HSV-2 and VZV (Figure 2B).

**TABLE 2:**
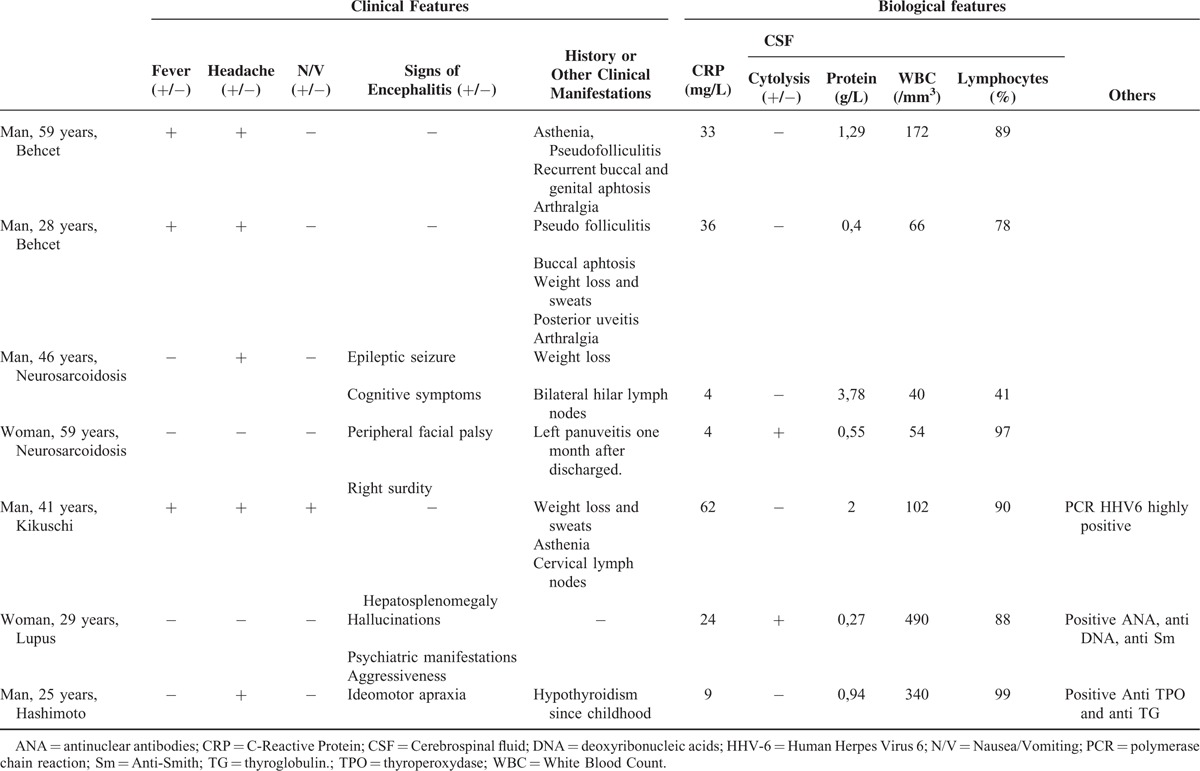
Clinical and Biological Characteristics of Patients With Inflammatory Disease

Three other patients had definitive diagnoses: acute disseminated encephalomyelitis in a 26-year-old man, carcinomatous meningitis from lung epithelial carcinoma in a 56-year-old man, and a cerebral venous thrombosis revealed by AM in a 16-year-old man.

### Therapeutic Management of Aseptic Meningitis With Uncertain Diagnosis

In 97 patients, the diagnosis was uncertain. PCR analysis for enterovirus, HSV-1, and VZV was lacking in 31, 16, 28 cases, respectively.

**FIGURE 3:**
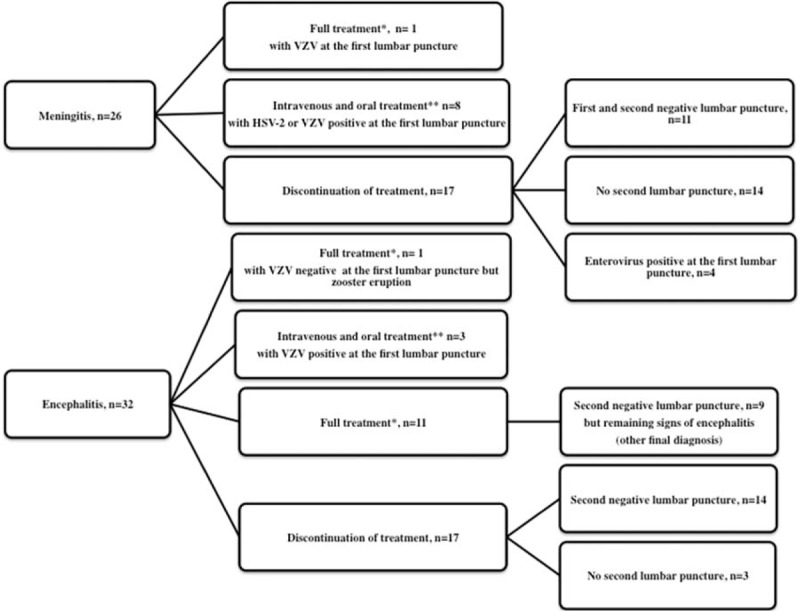
Outcome of acyclovir prescription in internal medicine department. Legends: ^∗^ full treatment = 14 to 21 days of intravenous acyclovir for HSV and 10 days for VZV; ^∗∗^intravenous and oral treatment = intravenous acyclovir 4 to 10 days followed by oral valaciclovir for an overall duration of 14 days.HSV = herpes simplex virus.

Twenty-six patients (26.8%) remained free of treatment, whereas antiviral or antibiotic therapy was initiated in the emergency department for the other 71 patients. Of these, most completed a treatment for 1 (or more) suspected etiologies (Table 3): 22 for listeria meningitis, 6 for bacterial meningitis, 14 for HSV or VZV encephalitis (Figure 3), 8 for tuberculosis, 9 for the suspicion of both listeria meningitis and other bacterial meningitis, and 7 were simultaneously given several treatments, as the etiology was uncertain. Ten patients were considered to have viral meningitis and did not complete a specific treatment.

**TABLE 3:**
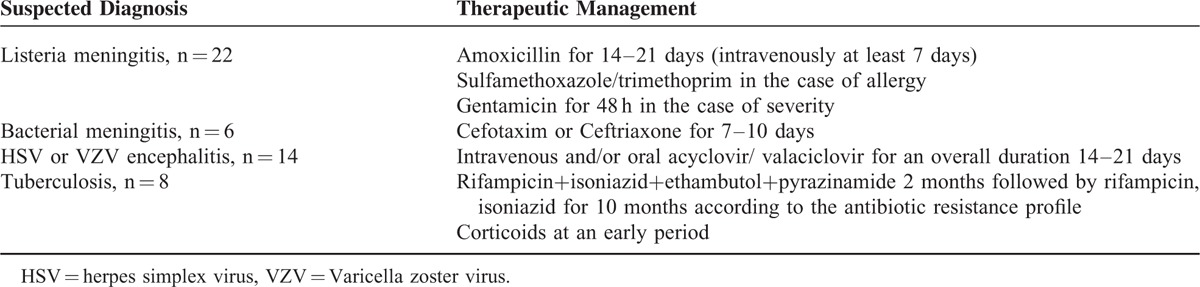
Therapeutic Management of Patients With Suspected Etiologies

Amoxicillin was more frequently administered to patients with fever and phono-photophobia (*P* = 0.03), or encephalitis (*P* = 0.025) and in the case of blood neutrophil count greater than 8000/mm^3^ or with proteinorachy over 1 g/L (*P* = 0.016). Cephalosporins were more frequently administered in patients with fever and when CSF WBC were >50% (*P* = 0.02).

Intravenous acyclovir was administered to 58 patients in the Emergency department, irrespective of the characteristics of the CSF, but more frequently to patients with encephalitis (*P* < 0.0001) or HSV-2 skin or genital infection (*P* = 0.04). The therapeutic management of these patients is described in Figure 3. In patients without encephalitis, treatment was mostly discontinued (17/26), sometimes after a second negative lumbar puncture. Among the 32 patients with encephalitis, the treatment was discontinued in 17, despite a second lumbar puncture. For some patients, the results of the first lumbar puncture were positive for VZV or HSV-2, and patients were given intravenous acyclovir few days, followed by valaciclovir by oral route for an overall duration of 14 days.

### Cases of Aseptic Meningitis in HIV-Infected Patients

HIV status was known in 156 patients and 23 were HIV-positive (14.7%). Meningitis led to the diagnosis of HIV infection in 5 cases (4 cases with a CD4+ T cell level <200/mm^3^). The etiological diagnosis of AM was definitive in only 6 cases (26%): 3 cases of VZV meningitis, 1 case of enterovirus meningitis, 1 case of cryptococcal meningitis, and 1 case of acute HIV meningitis. As for the other patients, 6 were treated for tuberculosis, 3 for HIV-related meningo-encephalitis, and others for listeria, syphilis, Lyme disease, cytomegalovirus, HSV. All had good outcomes except for 1 patient (CD4 = 6/mm^3^) who died despite several antibiotics and antiviral therapies. A post-mortem was not performed.

## DISCUSSION

To the best of our knowledge, this is the largest cohort of adult patients with aseptic meningitis (AM) reported to date in Internal Medicine. This retrospective study aimed to describe their etiologies, their clinical and biological pictures, and their therapeutic management.

We have shown that enteroviruses were the main causal agents, followed by HSV-2 and VZV. A systematic examination and analysis of CSF results can help distinguish them. The prevalence of inflammatory diseases in patients admitted to internal medicine for aseptic meningitis was not rare (4%). The high variability in CSF results among different inflammatory diseases makes clinical examination upon admission challenging.

An etiology was identified in 46.1% versus 66% of the cases in the cohort of Kupila et al. This was probably linked to the fact that they analyzed more types of fluids and that their study was prospective.^1,15^ Other authors have reported a higher percentage of unexplained cases.^2^ Incomplete PCR analysis upon admission to the Internal Medicine Department may have contributed to the higher prevalence of unexplained cases. This is a common issue, as performing a second lumbar puncture may be considered too invasive for a rapidly recovering patient. Therefore, all patients considered to have “probable viral meningitis” refused a second lumbar puncture. Positive PCR tests for enterovirus in the emergency room could have spared a patient with mild symptoms from an unnecessary hospital admission.

Enteroviruses were the main causal agents (20% of AM), followed by HSV-2 (8.3%) and VZV (6.1%), in close proportions to those previously reported.^1,2^ As opposed to headache and neck stiffness, the frequency of nausea or vomiting and phono-photophobia was higher when meningitis was due to HSV-2, in comparison with meningitis due to enteroviruses and VZV, and in accordance with a previous study.^3^ Due to the high prevalence of simultaneous genital herpes infection (36%) in cases of meningitis due to HSV-2 in our study, in comparison with others (8.7–25%), examination of external genital organs should be more systematic at admission of patients presenting with symptoms of meningitis.^3,16,17^ The frequency of encephalitis due to VZV coincided with previous studies.^1,18^ We found a lower frequency of skin manifestations (33.3%) than other studies, probably due to the young age of our patients.^1,18^ Moreover, low protein levels (<0.7 g/L approximately, Figure 2) and low lymphocyte percentage (<80% approximately, Figure 2) in CSF are strong arguments for enterovirus meningitis. Conversely, distinguishing between meningitis due to HSV-2 and VZV on CSF results remains difficult. A complete and systematic examination can help distinguish these etiologies. It guides the treatment and the discussion of a second lumbar puncture, especially when PCR analysis is lacking upon admission to Internal Medicine. Additionally, if the anamnesis suggests a recent respiratory infection in the patients or their contacts, respiratory tract samples can be useful to avoid a second lumbar puncture and to reduce the prevalence of meningitis with uncertain diagnosis.^1,15^

The investigations based upon the clinical picture at admission and the outcome allowed the diagnosis of 20 nonviral AM. Inflammatory diseases accounted for 4% of AM and were inaugural. Although AM is a well-identified complication in patients with inflammatory diseases, it remains a rare event. The frequency of CNS involvement in patients is 17% in Behcet disease,^19,20^ roughly 5% in sarcoïdois,^21^ <0.001% in Hashimoto disease and 10% in Kikuchi disease.^22,23^ Meningitis is observed in ∼1.6% of patients with Systemic lupus erythematosus, but a microorganism is frequently identified (60%) as they are at an increased risk of infection.^24^ The CSF lymphocyte count in inflammatory diseases approaches that observed in meningitis due to enteroviruses, whereas the mean lymphocyte percentage and the mean protein level were close to those found during meningitis due to HSV-2 and VZV (Figure 2). However, we observe a high variability in CSF results among different inflammatory diseases and even in patients with the same inflammatory disease, in accordance with retrospective case series.^20,24–26^ In inflammatory diseases, higher prevalence of encephalitis is observed but CSF findings broadly overlap with viral meningitis, making clinical examination upon admission challenging.

No specific guidelines for aseptic meningitis were followed during the study period, except in case of encephalitis, and the management varied from patient to patient as can be seen from acyclovir or antibiotic prescriptions.^14^ Even so, clinical outcome was favorable in 179/180 patients. In recent years, a tendency has developed among many physicians to start intravenous acyclovir as well as broad-spectrum antibiotics, even though there is no evidence base for their usage. As the features of meningitis are nonspecific, clinical suspicion of bacterial meningitis, HSV or VZV leads to administration of antibiotics or acyclovir within few hours of admission to the emergency unit even in the absence of encephalitis, whereas waiting for the results of further diagnostic investigations, especially PCR analysis. In our study, antibiotics and antiviral therapy were clearly linked to the severity of the disease. The common problem is the decision about when to stop treatment, especially in patients who appear to be recovering and who have not been fully investigated. In most cases, the treatment was not completed, but the rationale and modalities discontinuing or pursuing were unclear. Concerning acyclovir prescription, repeating the lumbar puncture in the case of encephalitis to ensure that the CSF is negative for HSV before stopping was mostly respected.^15,27^ Either way, unwarranted prolonged hospitalizations and costly intravenous therapy should not be preferred to a supplementary lumbar puncture in patients with encephalitis. The PCR remains positive for several days after starting acyclovir treatment and can become positive whereas the first was not.^15,16,27^ Concerning the antibiotics, the 16S rRNA sequencing may represent an interesting tool to avoid or stop the antibiotics when decapitated meningitis are suspected, especially when the degree of clinical suspicion is low.^28^

We noticed that the therapeutic management of HSV-2 and VZV meningitis was inappropriate. Compared to HSV-1 encephalitis, the outcome of HSV-2 or VZV meningitis in adults is usually self-limited, with spontaneous recovery.^15,16^ The impact of antiviral treatment in the normal host, including shortening symptomatic meningitis, reducing morbidity, or preventing recurrence, has not been established, as opposed to immunocompromised patients.^16,29^

Finally, the high frequency of HIV-positive patients (14.7%) among patients with AM reflects the local recruitment. Tuberculous meningitis is recognized as a common complication in HIV-positive patients.^30,31^ AM due to VZV and enteroviruses was diagnosed in 4 HIV-positive patients. An HIV test should be performed for all patients with encephalitis or with suspected encephalitis irrespective of the interpretation of possible risk factors.^15^ Moreover, the numerous and simultaneous pathogens in HIV-positive patients justify performing a complete biological investigation at their admission.^32,33^

The following limitations of our study should be mentioned: its retrospective nature resulting in missing data and in the variability of the therapeutic management. Viral PCR was not systematically performed in the emergency department, and our study may have underestimated the prevalence of enterovirus meningitis. Moreover, as we aimed to report our experience in an Internal Medicine Department, patients with meningitis that was immediately considered as viral meningitis in the Emergency Department were not included in this study, making the prevalence of others diagnoses higher in Internal Medicine Department than its true value in other settings. Finally, the retrospective analysis makes the diagnosis of drug-induced meningitis difficult. Despite the number of cases reported, it is difficult to propose a tree of explorations for the management of AM regarding the retrospective nature of our study. In addition, due to the variability in the presentation of AM even for a given etiology, it is difficult to definitely exclude a diagnosis on few clinical or biological data. The clinicians have to keep in mind the large number of etiologies, from the viral meningitis to the anti-*N*-methyl D-aspartate receptor encephalitis.

In conclusion, we have shown that the prevalence of inflammatory diseases in patients admitted to internal medicine for aseptic meningitis is not rare (4%). The PCR upon admission to the Emergency Department is obviously of major importance for optimizing therapy and management as soon as possible, but meningitis due to viral agents or to inflammatory diseases could also be distinguished according to several clinical and biological characteristics. As recommendations are now available concerning the prescription of antiviral agents in viral meningitis, better therapeutic management is expected in the future.
